# Addressing rainfall data selection uncertainty using connections between rainfall and streamflow

**DOI:** 10.1038/s41598-017-00128-5

**Published:** 2017-03-16

**Authors:** Morgan C. Levy, Avery Cohn, Alan Vaz Lopes, Sally E. Thompson

**Affiliations:** 1 Energy and Resources Group, University of California, Berkeley, USA; 2 Fletcher School, Tufts University, Medford, USA; 3 National Water Agency (ANA), Brasilia, Brazil; 4 Department of Civil and Environmental Engineering, University of California, Berkeley, USA

## Abstract

Studies of the hydroclimate at regional scales rely on spatial rainfall data products, derived from remotely-sensed (RS) and *in*-*situ* (IS, rain gauge) observations. Because regional rainfall cannot be directly measured, spatial data products are biased. These biases pose a source of uncertainty in environmental analyses, attributable to the choices made by data-users in selecting a representation of rainfall. We use the rainforest-savanna transition region in Brazil to show differences in the statistics describing rainfall across nine RS and interpolated-IS daily rainfall datasets covering the period of 1998–2013. These differences propagate into estimates of temporal trends in monthly rainfall and descriptive hydroclimate indices. Rainfall trends from different datasets are inconsistent at river basin scales, and the magnitude of index differences is comparable to the estimated bias in global climate model projections. To address this uncertainty, we evaluate the correspondence of different rainfall datasets with streamflow from 89 river basins. We demonstrate that direct empirical comparisons between rainfall and streamflow provide a method for evaluating rainfall dataset performance across multiple areal (basin) units. These results highlight the need for users of rainfall datasets to quantify this “data selection uncertainty” problem, and either justify data use choices, or report the uncertainty in derived results.

## Introduction

Quantifying precipitation patterns at regional scales is essential for water security^1, 2^, but is compromised by discrepancies in rainfall datasets^3–5^. Spatial rainfall data products have proliferated, drawing on differing information sources, using different techniques to impute that information through space, and varying in their spatial extent and spatio-temporal resolution^6^. The proliferation of such rainfall datasets facilitates applied research at regional spatial scales, but raises the risk that naïve use of an individual rainfall product may introduce bias into subsequent analyses, relative to the full range of representations of the rainfall field available^7^. Addressing this risk requires quantifying the differences between available rainfall data products, and, if possible, identifying and working with only those datasets that are most suitable for the intended analysis. Here we firstly show that the differences across daily rainfall datasets, for a test case in Northern Brazil, are large enough to require such uncertainty characterization. Next we demonstrate that comparison of datasets with a mechanistically related, but independently observed environmental variable, in this case streamflow, can provide a basis for selecting among available rainfall products. Although our proximate goal is to identify and reduce the uncertainties associated with naïve selection of a rainfall data product for hydrologic purposes, the approach is generalizable to other climatic products and applications.

Regional rainfall data are collected through remote sensing (RS) and *in*-*situ* (IS) rain gauge observations. At regional scales, and in remote, rural or developing regions, the rainfall data products generally available and most applicable for hydroclimatological analyses^4^ are based on RS data, IS data, or both. IS data provide precision and accuracy at a point, but are often distributed sparsely and heterogeneously in space, and discontinuously in time^8, 9^, and may pose quality control challenges^10, 11^. RS data have consistent coverage and represent spatial heterogeneity, but are often biased, with uncertainties that are dependent on topography, climate, and the level of spatial and temporal aggregation^3, 5, 12^. Differences between rainfall datasets emerge, especially at daily or sub-daily temporal resolutions^7^, mostly due to artifacts introduced during data processing. For RS data, such artifacts can include a combination of satellite data retrieval technologies and associated processing algorithms, as well as IS calibration sources and methods^4^. For IS data, artifacts may derive from gauge measurement quality, availability, and the imputation and/or interpolation methods used^13–15^. While RS data may be a preferred alternative to IS data in settings with sparse rain gauge networks^16^, at regional scales, both data types, and their spatial imputations, are expected to differ from ‘true’ (and unknown) rainfall fields.

Consequently, the challenge of data selection given the uncertainty associated with datasets is not to determine the ‘most accurate’ dataset, for which there is no universal assessment^4, 17^, but instead to quantify the uncertainty in any given analysis that derives from the different representations of reality by the available ensemble of data products. If possible, data selection should also identify the most ‘fit-for-purpose’ dataset, based on its fidelity to the features of rainfall (e.g. mean, extremes, trends, or correspondence with an independently measured and mechanistically related environmental variable) most pertinent to a given study topic.

**Figure 1:**
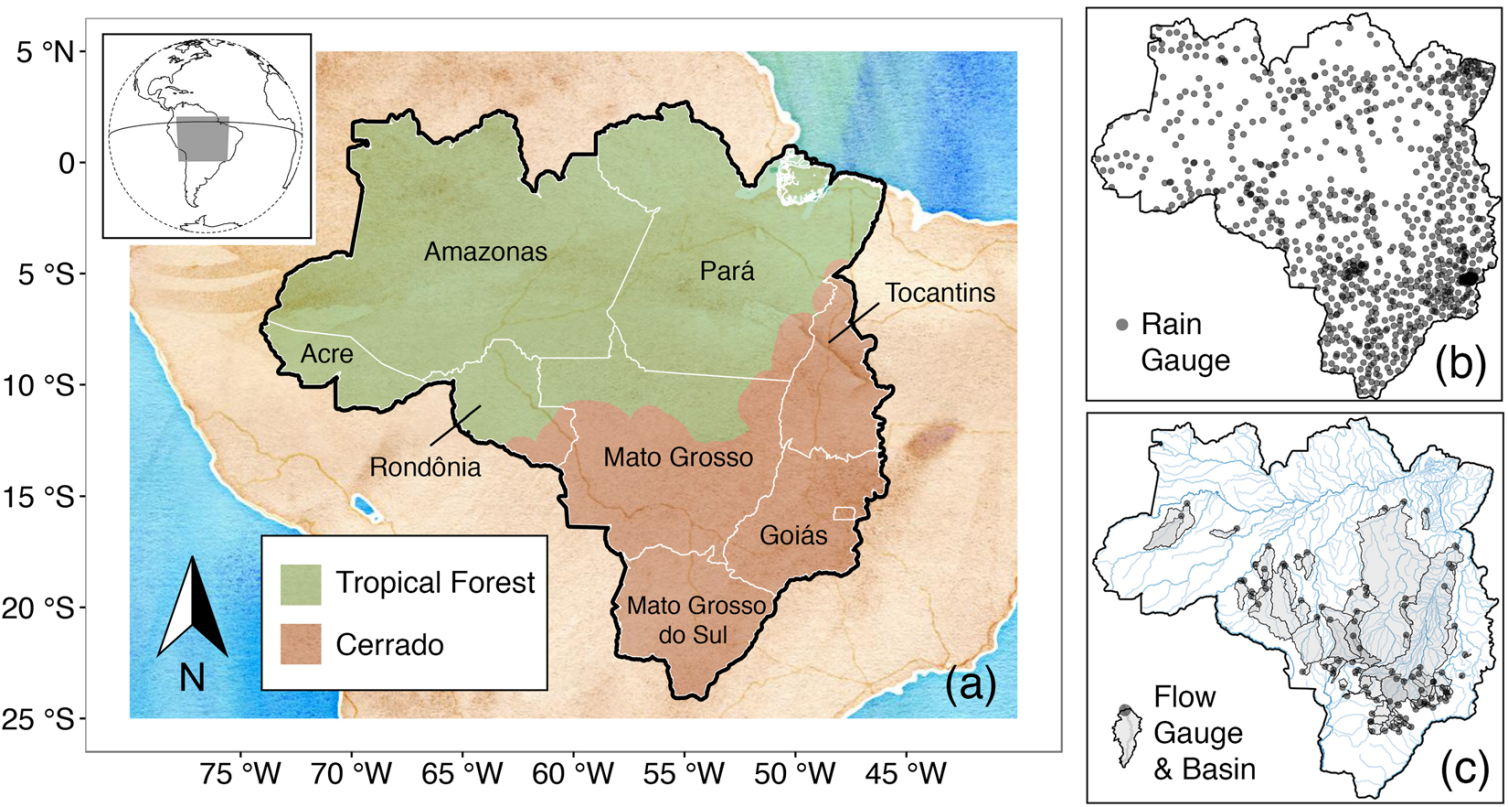
Study region and locations of *in*-*situ* (IS) rainfall and streamflow gauges. Panel (a) shows the Amazon-Cerrado transition states of Brazil. Panels (b,c) show the location of 942 rain gauges and 89 streamflow gauges and associated catchment areas (river basins) used in the analysis. The majority of the river basins in (c) drain to the north, and are headwater basins of the Amazon and Araguaia-Tocantins River Basins; basins located in the south are headwater basins of the Paraguay and Paraná River basins. These maps were generated in R, Version 3 (https://cran.r-project.org/)^76^ using data from the curated data package accompanying this manuscript^30^ and biome boundary data^77^.

Our case study region, the rainforest-savanna (Amazon-Cerrado) transition zone in Brazil (Fig. 1(a)) has experienced dramatic changes in land-cover, with anticipated feedbacks to regional climate^18, 19^, and thus to the wide variety of rainfall-dependent ecosystem services provided in the region, including agricultural industries, the hydropower sector^20, 21^, and extensive regional forest. Variability and change in the Amazon and surrounding region’s precipitation therefore affect Brazilian economic, food, and energy security, and potentially also the health of the Amazon rainforest and the global climate system^22–24^. Rainfall in center-west and northern Brazil is monitored through a relatively sparse rain gauge data network (15 or fewer rain gauges per 10^4^ km^2^), comparable to inland regions of South America; sub-Saharan Africa; and central, east, and southeast-Asia^25^. These low densities are likely to result in non-trivial differences between regional rainfall data products^26^ (in Switzerland, rain gauge densities of >24 rain gauges per 1,000 km^2^ were required to avoid density-dependent biases^9^).

Rainfall data in center-west and northern Brazil are therefore likely to be inaccurate at regional scales, yet remain highly relevant to a wide range of policy and planning efforts. For the purposes of this paper, we focus on the quantification of regional daily rainfall statistics needed for hydrologic analyses. Daily rainfall data, or statistical representations thereof, are needed as input to a broad range of hydrological models and empirical analyses that assess spatial or regional trends or drivers of flow variability^27, 28^. We analyze a suite of statistical descriptors of daily rainfall, including the daily mean rainfall depth, wet-day mean rainfall depth, and percent occurrence of wet days. These all influence streamflow response^29^, and are referred to as “rainfall characteristics” in the remainder of this paper (results for a more expansive range of rainfall statistics are also presented as Supplementary Information).

The rainfall datasets used in this analysis (Table 1) include four global and quasi-global gridded (RS and IS) products, and five custom interpolations of the Amazon-Cerrado rain gauge network, containing 942 gauges (Fig. 1(b)) and managed by the Brazilian government water management agency (Agência Nacional de Águas - ANA). The curated IS rainfall and streamflow data used in this analysis are provided in a data package: “Curated rain and flow data for the Brazilian rainforest-savanna transition zone”^30^. We interpolated each day’s set of reporting rain gauges over a 16 year period, from January 1, 1998 to December 31, 2013, using five interpolation methods ranging from a naïve nearest-neighbor to more sophisticated geostatistical approaches (see Methods).

**Table 1:** Daily rainfall datasets.

Category	Name	Description	Type	Resolution
Gridded	GPCP	Global Precipitation Climatology Project (GPCP), Version 1.2	RS, IS	1°
	CPC	Climate Prediction Center (CPC) Unified Gauge-Based Analysis of Global Daily Precipitation, Version 1 and RT	IS	0.5°
	TRMM	Tropical Rainfall Measuring Mission (TRMM) 3B42, Version 7	RS, IS	0.25°
	PERSIANN	Precipitation Estimation from Remotely Sensed Information using Artificial Neural Networks - Climate Data Record (PERSIANN-CDR), Version 1.1	RS, IS	0.25°
Custom	UKP	Universal Kriging with PERSIANN (predictors: elevation, latitude, longitude, and PERSIANN-CDR)	IS, RS	0.25°
	UK	Universal Kriging (predictors: elevation, latitude, longitude)	IS	0.25°
	OK	Ordinary Kriging	IS	0.25°
	IDW	Inverse Distance Weighting	IS	0.25°
	VP	Voronoi (or Thiessen) Polygons	IS	0.25°

Gridded data are global and quasi-global products; custom data are regional (local) interpolations of IS rainfall data obtained from Brazil’s Agência Nacional de Águas (ANA). See Methods for sources and additional details.

Intercomparison of these products is not straightforward. Point (IS) estimates of rainfall are not directly comparable with gridded (RS) estimates^31, 32^. Because streamflow responses arise at river-basin scales, we focus here on an intercomparison at spatially averaged river-basin domains, calculating the rainfall characteristics over 89 river basins in the study region (Fig. 1(c)), as well as on a 0.25° resolution grid. Given this focus, and the characteristics of the region and its rainfall, we might expect gridded RS products to be preferred. RS products are often preferred over IS products in regions where low gauge density prohibits high quality interpolation^9^, and the flat, low-altitude, and moderately wet conditions in central and northern Brazil are considered optimal for RS rainfall retrieval^3, 5, 33, 34^.

Our approach to data selection and quantification of uncertainty involves an initial intercomparison of the rainfall characteristics, at grid and basin scales, across the nine rainfall datasets. In the absence of an independent set of empirical measurements against which to compare the datasets, the resulting range in the rainfall characteristics across datasets provides an ensemble measure of the uncertainty associated with these characteristics, which we measure using the maximum absolute deviation (MAD) and standard deviation across datasets for each statistical measure in each basin. To illustrate how such dataset differences may propagate into subsequent analyses, we compute several hydroclimatic indices or analytical results - the runoff ratio (ratio of annual runoff to rainfall), the evaporation ratio (ratio of annual evapotranspiration to rainfall), the Horton index^35^ (ratio of evapotranspiration to available soil water), and long-term (inter-annual) trends in daily rainfall, evaluated on monthly timescales for each basin, and again compute MAD and the standard deviation for each basin. The range in these computed indices and trends provides an ensemble description of hydroclimatic uncertainty due to the propagation of data selection uncertainty into these simple analytical outputs.

Having demonstrated that the differences in rainfall characteristics and their propagation into simple analyses are large enough to cause concern, we next attempt to select a rainfall dataset for use in hydrologic studies, by the approach of comparing rainfall datasets to an independently measured, but mechanistically related, environmental variable. In this case, we use streamflow records across 89 river basins to provide such an independent metric. Given the mechanistic connection between streamflow and rainfall, whereby preceding rainfall events drive subsequent streamflow increases, we use measures of time series correspondence or similarity between daily rainfall (at river basin scales) and streamflow for this intercomparison. Specifically, we treat datasets that maximize the correlation between rainfall and streamflow timeseries, and the correspondence of rainfall with streamflow peaks (see Methods), as being the most informative for hydrologic studies.

## Results

### Rainfall characteristics

**Figure 2:**
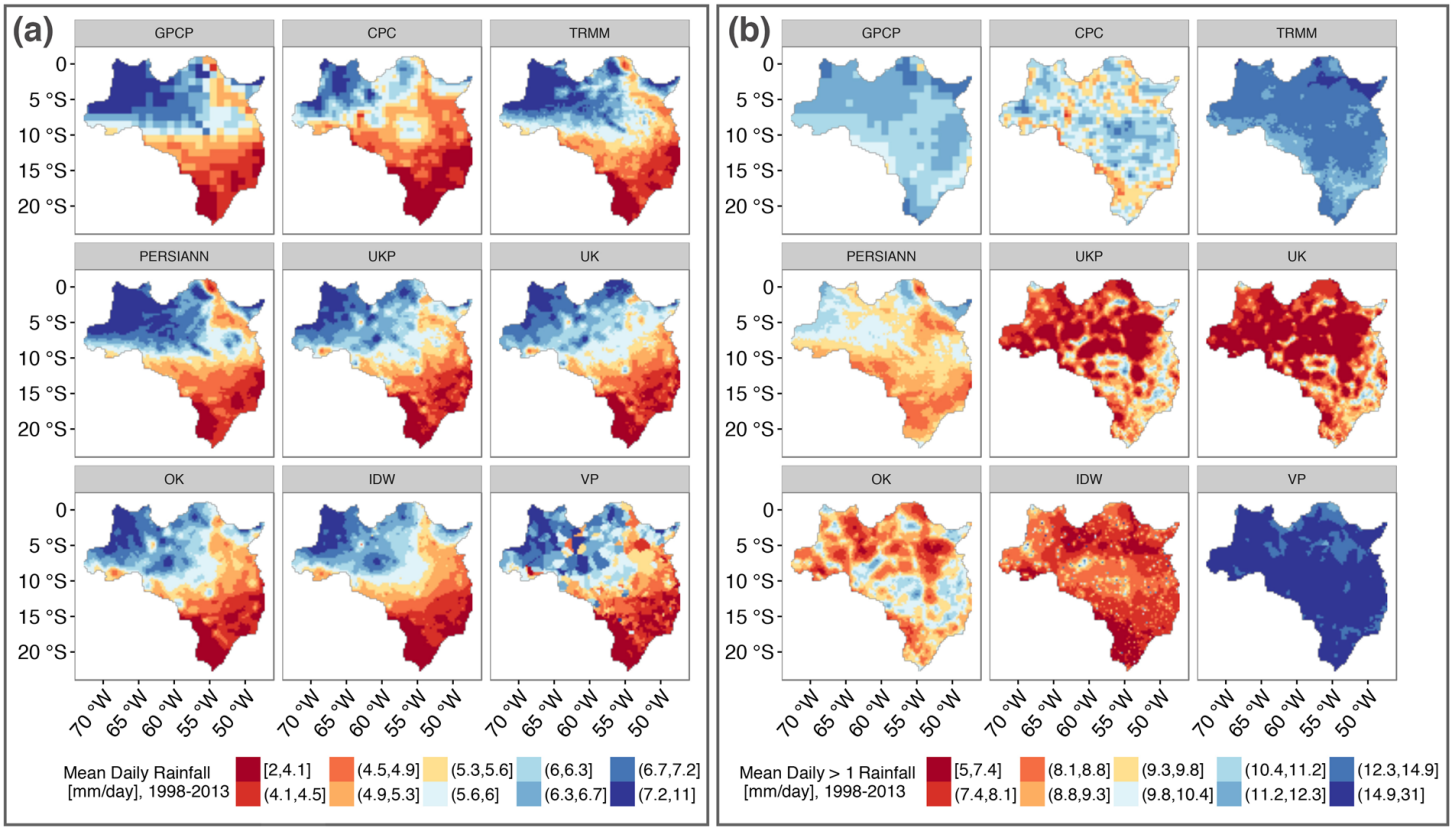
Spatial variation in the representation of descriptive statistics by different rainfall datasets. Panel (a) shows mean daily rainfall (depths in mm/day), and panel (b) shows mean wet-day rainfall (depths in mm/day for days with ≥1 mm/day). Both statistics were calculated at each 0.25° resolution grid cell in the study region using all daily data between 1998–2013. See Table 1 and Methods for dataset details. GPCP, TRMM, and PERSIANN are gridded datasets comprised of RS and IS data sources; CPC is a gridded dataset comprised of IS data; UKP is a custom interpolation of IS and RS (PERSIANN) data sources; UK, OK, IDW, and VP are custom interpolations of IS data. These maps were generated in R, Version 3 (https://cran.r-project.org/)^76^.

Figure 2(a) shows mean daily rainfall over the study period (1998–2013) at individual grid cells for all nine rainfall datasets, demonstrating relative consistency in large-scale spatial patterns and magnitudes of rainfall, although the mean at individual locations can differ substantially. Figure 2(b), however, demonstrates dramatic differences in the representation of wet-day (≥1 mm/day) rainfall. This illustrates that rainfall detection, to which RS data errors are principally attributed^3^, and representation of extremes, which vary with the level of spatial aggregation and due to interpolation method^13, 14^, differentiate datasets. Differences across datasets - in spatial patterns and magnitudes - persist across a suite of other statistics (see Supplementary Figures 2–5, which depict grid-cell-level median and wet-day median rainfall depths, maximum and standard deviation of rainfall depths, mean annual total rainfall, and wet-day occurrence of rainfall). Figure 2 shows point-estimates of rainfall properties at individual grid cells. However, we are primarily concerned with observations of area-integrated rainfall, and the remaining results pertain to areal spatial units (either sample areas or river basins, as noted).

**Figure 3:**
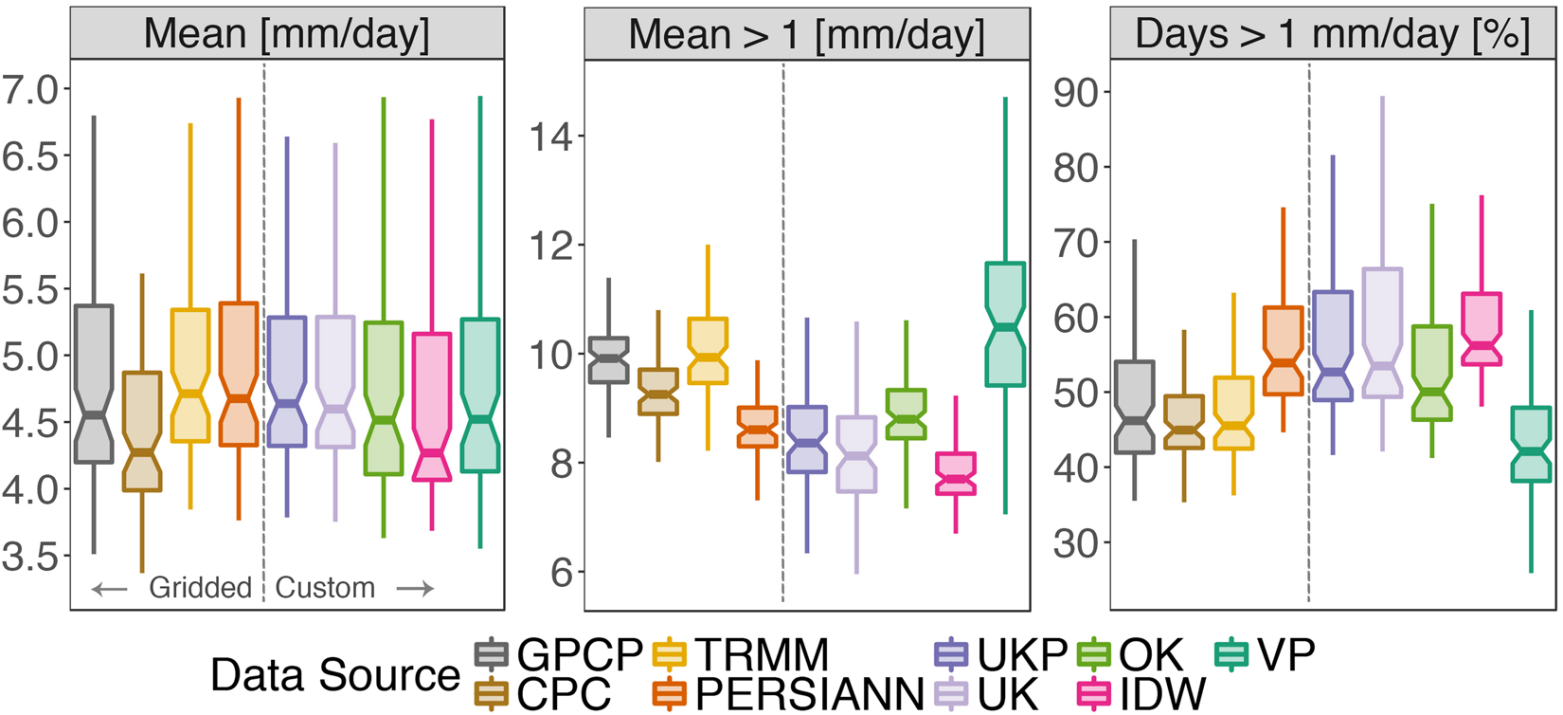
Daily rainfall statistics in river basins according to different rainfall datasets. From left to right, respectively, the panels show the simple daily mean, wet-day (≥1 mm/day) mean, and wet day occurrence (percent of days with ≥1 mm/day) of rainfall for 89 river basins. Each boxplot is generated with n = 89 (river basin) statistic results, calculated using basin area-average rainfall from the given rainfall dataset (colors) from all days between 1998–2013. Outliers are not shown. The vertical dashed line separates gridded from custom datasets (see Table 1).

Figure 3 shows variations in the same statistics as presented in Fig. 2 - the mean and wet-day mean, as well as the occurrence of wet days, over river basin units of analysis (Supplementary Figure 6 shows basin-level percentiles, mean annual total, standard deviation, and maximum). Again, there is overlap in the mean daily rainfall estimates, but significant variation exists in wet-day mean values across rainfall datasets. These results suggest that the rainfall datasets can be divided into two groups: the first (I) includes the gridded datasets GPCP (RS), CPC (IS), and TRMM (RS), and the nearest-neighbor interpolation VP (IS); and the second group (II) includes the remaining interpolations UKP (IS, RS), UK (IS), OK (IS), and IDW (IS), and the gridded PERSIANN (RS) dataset. Figure 3 shows that group II datasets report a greater number of wet days, but lower mean rainfall on those wet days, relative to group I. The lower mean wet-day rainfall of group II stems from the fact that group I data report more wet-day extremes (see Supplementary Figures 3 and 6), which upwardly bias the mean wet-day rainfall of group I, despite those data showing fewer wet days. While group I *wet*-*day* medians are also greater than group II in accordance with wet-day means, group I *all*-*day* medians are *less* than those of group II (see Supplementary Figures 2 and 6). This is due to the combination of greater wet-day occurrence and medium-intensity rainfall (1–10 mm/day) in group II (see Supplementary Figure 7). These differences persist across the range of rain gauge densities in the study region (see Supplementary Figure 7).

Greater wet-day occurrence in group II custom interpolations (UKP, UK, OK, and IDW) likely results from greater rates of local detection of medium intensity rainfall by rain gauges relative to satellite sources, combined with spatial smoothing of those rainfall events. In the case of PERSIANN, elevated wet-day occurrence can be attributed to a combination of the rainfall estimation algorithm and/or incorporation of multiple RS and IS rainfall products that are unique to this RS dataset compared to earlier RS products (GPCP, CPC, TRMM)^36^. In summary, divergent features of the group I datasets (lower wet-day occurrence and medium intensity rainfall depths, greater extremes), and group II datasets (greater wet-day occurrence and medium intensity rainfall depths, lower extremes) may result in similar mean daily average values across large regions as shown in Fig. 2(a). Thus, there may be consistency across rainfall datasets in analyses relying upon regional mean daily rainfall values (only). However, different datasets will propagate significant uncertainty into analyses relying on estimation of wet-day rainfall occurrence or depths, quantiles, and extremes. These findings are consistent with previous assessments of interpolated and gridded environmental data^13, 14, 37^.

Calculation of the maximum absolute deviation (MAD) between any two datasets’ area-average rainfall (averages over areas of the 0.25° grid) provides a simple quantification of dataset divergence and thus the range of the data ensemble. We calculated 1998–2013 MAD at daily, monthly, and annual time scales at 100 regularly-sampled locations, for areas ranging from large to small (circles with radii of 200 km and 10 km, centered at the same 100 locations). This sample design accounts for the fact that different regions, and differently-sized sample units, have different rain gauge densities. At a daily resolution, the mean (median) MAD between any two datasets’ area-average rainfall is 7–12 mm (5–8 mm); at a monthly resolution, it is 56–97 mm (46–82 mm); at an annual resolution, it is 372–576 mm (310–497 mm). The ranges are from statistics calculated for the large to small sample units, respectively. These differences are comparable to global climate model (GCM) biases: projections from the Coupled Model Intercomparison Projects Phase 5 (CMIP5)^38^ have annual biases relative to a single rainfall data product of −25% (approximately −250 to −550 mm/year) in northern Brazil^39^, indicating that selection of a different rainfall dataset for reference has the capacity (at an extreme) to either eliminate or double the estimated model bias.

### Trends and Hydroclimate Indices

**Figure 4:**
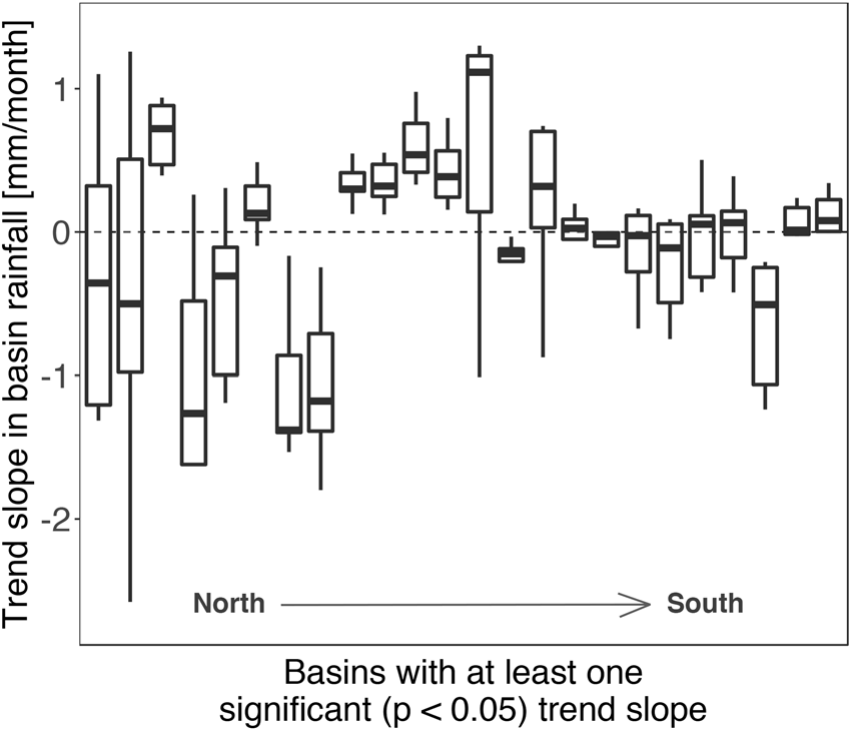
River basin monthly total rainfall trend slope variation across rainfall datasets. Each boxplot shows the distribution of individual basin trend slope coefficients (excluding outliers) estimated using nine rainfall datasets; the 24 basins shown are those for which at least one rainfall dataset has a significant (p < 0.05) trend; not all trend slopes are significant. Basin boxplots are ordered left to right according to the latitude of the basin centroid.

Evaluation of hydroclimatic indices and temporal rainfall trends demonstrates the propagation of rainfall data selection uncertainty into a standard analysis. Although temporal trend analysis is not especially meaningful over a 16-year time period, it demonstrates the potential for trend detection and attribution to be amplified or eliminated by data uncertainty. We calculated monotonic trend slopes (corrected for monthly correlation) and associated p-values for total rainfall by month for all 89 river basin in the study region between 1998–2013 (see Methods). Variation in the estimated trend slopes for basins where at least one rainfall dataset had a statistically significant trend are shown in Fig. 4. Trend slopes, particularly for basins in the north of the study region where rain gauges are especially sparse, do not agree across rainfall datasets. Rainfall datasets agree on the sign of the slope in only eight of the 24 basins (four basins with all positive slopes, and four basins with all negative slopes).

**Figure 5:**
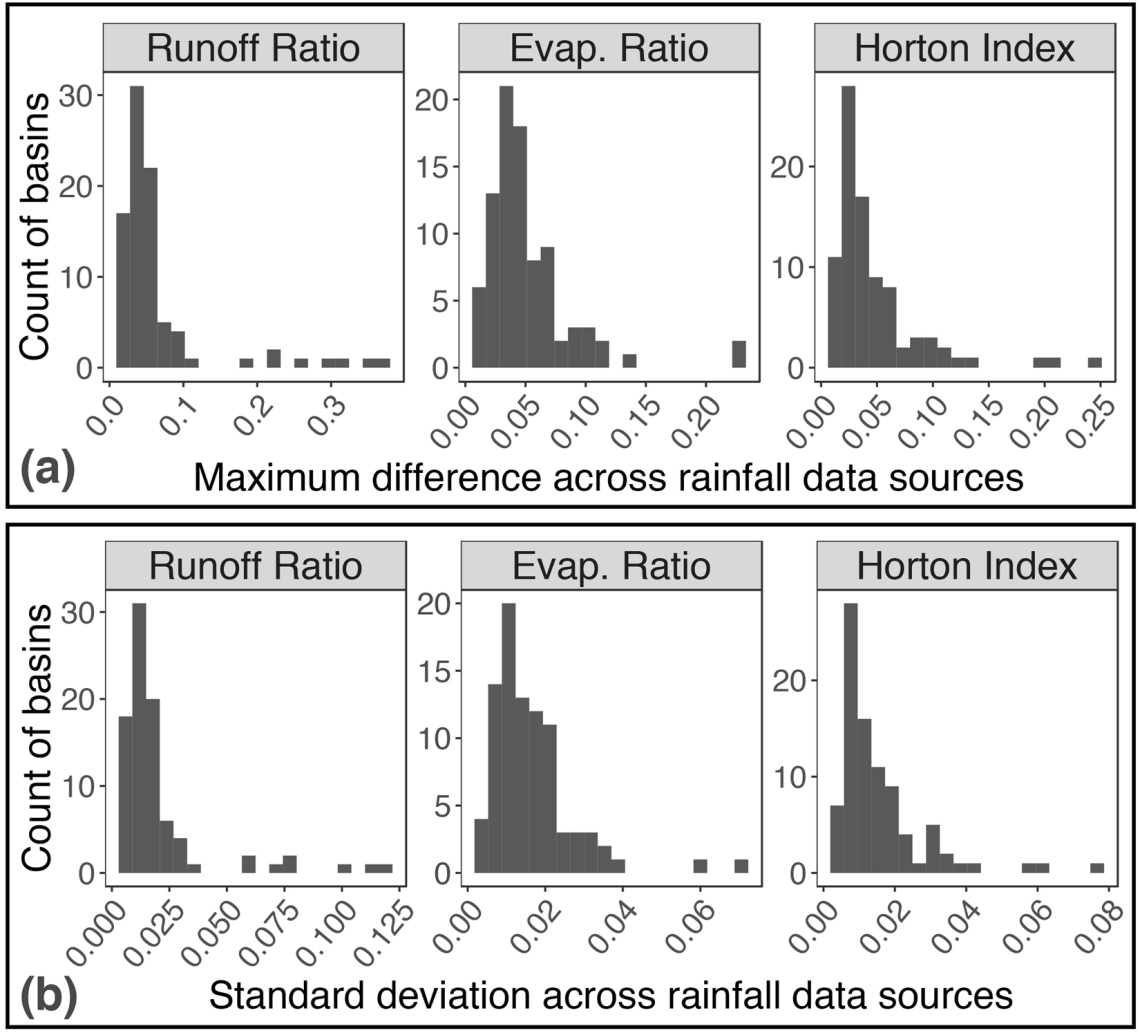
Distribution of river basin hydroclimate index differences and variance across rainfall datasets. Panel (a) displays histograms of maximum absolute deviations between individual river basin hydroclimate indices calculated using each of the nine rainfall datasets. Panel (b) displays histograms of the standard deviation of individual river basin hydroclimate indices across the nine rainfall datasets. The runoff ratio is the runoff fraction of rainfall; the evaporation ratio is the evapotranspiration fraction of rainfall; and the Horton index is the evapotranspiration fraction of available soil water (see Methods for details). All index values range between zero and one.

The propagation of rainfall data selection uncertainty is further illustrated by hydroclimatic index measurements made using the different rainfall datasets. Hydroclimatic indices provide information on the relationships between climate, land use, and hydrology, which are critical to the examination of land use and climate change^35, 40^. They are estimated using both rainfall and streamflow at river basin scales (see Methods). The runoff ratio is the fraction of rainfall discharged from a river basin as streamflow (as opposed to evaporated or transpired at the land surface, or percolated to deep groundwater); the evaporation ratio complements the runoff ratio - it is the fraction of rainfall evapotranspired (as opposed to discharged or percolated); the Horton index compares evapotranspiration to soil water stores (as opposed to total rainfall). The mean (median) maximum absolute deviation between basin-level index values generated using any of the nine rainfall datasets (see Fig. 5(a)) is 0.05 (0.04) for the evaporation ratio and Horton index, and 0.06 (0.04) for the runoff ratio; the difference exceeds 0.25 - a quarter of the entire index range - for some basins. Similarly, the mean (median) standard deviation of basin-level index values (see Fig. 5(b)) is 0.02 (0.01) for all three indices; and can exceed 0.05 in some basins. Streamflow data is the same for all calculations within each basin, so these results demonstrate the sensitivity of basin-scale analyses to rainfall input data alone.

In the absence of information on a ‘best’ rainfall data source, and knowing that data selection uncertainty will propagate into analyses as demonstrated in Figs 4 and 5, distributions of index values obtained from multiple rainfall datasets can be used to quantify data selection uncertainty. For example, the mean of the standard deviations across all basins for a given index (e.g. mean of values shown in each panel of Fig. 5(b)) may be treated as an index- and region-specific standard deviation (*s*) attributable to rainfall data selection uncertainty. According to our analysis, in rainforest-savanna transitional Brazil, *s* is approximately 0.02 for all three indices. A straightforward confidence interval for the mean of index values obtained using the nine rainfall datasets over an *individual* basin ($\bar{x}$) in our study region is: $CI=\bar{x}\pm {z}^{\ast }S{E}_{\bar{x}}$, where $S{E}_{\bar{x}}=s/\sqrt{89}=0.002$ (see Supplementary Discussion for further details).

### Rainfall and Streamflow Correspondence

Figures 2, 3, 4 and 5 demonstrate the need for a procedure to guide rainfall data choice prior to conducting analyses. We build on the precedent for evaluating rainfall data quality using the correspondence between rainfall and river flow^16, 33, 41^ by measuring the empirical correspondence between rainfall and streamflow records using two performance statistics: non-parametric Spearman’s rank correlation, and peak correspondence - the rate at which distinct rainfall peaks correspond to distinct flow peaks within a basin-specific response time window (see Methods). Streamflow rises and peaks in unregulated, rain-fed rivers are caused by preceding rainfall events in the rivers’ catchment, so the correspondence between appropriately-lagged and basin-integrated rainfall, and basin streamflow, measures a rainfall dataset’s ability to capture area-integrated rainfall patterns.

In validation tests, rainfall data from seven Australian river basins were randomly perturbed using additive noise, and true and perturbed rainfall datasets were evaluated relative to streamflow using the performance statistics. Both performance statistics identify the correct rainfall dataset 100% of the time when the random noise is equivalent to or greater than basin rainfall standard deviation. In cases where random noise is less than or equal to half the basin rainfall standard deviation (when differences between datasets are small), correlation still identifies the correct rainfall dataset 100% of the time, however peak correspondence identifies the correct dataset on average (across the seven test basins) 79% of the time or less (see Supplementary Discussion for details). Specifically, peak correspondence performs perfectly (100% correct identification) in some basins, but not others, when the signal to noise ratio is low. This is likely due to peak correspondence’s reliance on quick (storm) runoff response signatures (see Methods), which may vary in quality across different basins. In the study region, the 1998–2013 average maximum absolute deviation (MAD) between any two rainfall datasets on a daily time scale is 7–12 mm; the range of grid cell-level (temporal) standard deviations averaged across the study region for each individual rainfall datasets is between 7–13 mm. Thus, in the study region, individual rainfall dataset variation is on the order of variation between datasets, indicating that peak correspondence will perform as well or nearly as well as correlation in identifying datasets with greatest correspondence to flow. This is confirmed by the similarity in results from both statistics for the Brazilian data.

**Figure 6:**
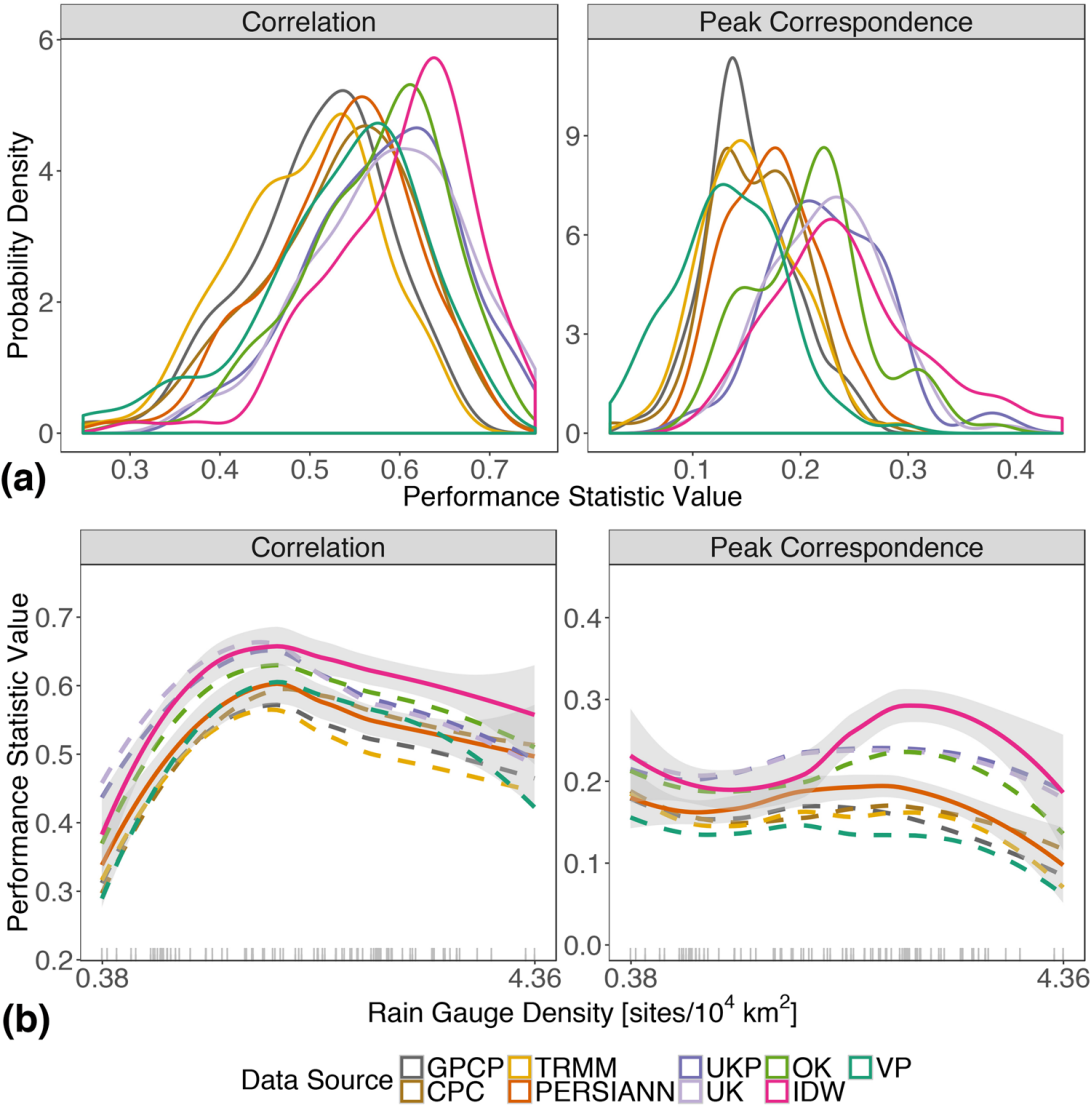
Differences in rainfall data quality as indicated by performance statistics measuring correspondence with streamflow. Panel (a) shows kernel-smoothed empirical probability distributions of performance statistics (correlation and peak correspondence) by rainfall dataset. Panel (b) shows the same performance statistics plotted as local regression-smoothed curves across the range of rain gauge densities in the study region, with 95% uncertainty intervals (shaded). In (b), solid lines and shaded regions indicate the best-performing gridded (PERSIANN) and custom-interpolated (IDW) datasets and their 95% uncertainty intervals, respectively; non-overlapping uncertainty intervals indicate distinguishable performance between datasets; dashed-lines indicate all other datasets with uncertainty intervals that are not displayed, but are of similar width; gray tick-marks at the bottom illustrate the spread of rain gauge densities in the 89 river basins.

Figure 6 presents distributions of the performance statistics in 89 river basins (panel a), and illustrates the sensitivity of the performance statistics to rain gauge density within the river basin (panel b). The better the performance of the dataset, the farther to the right are the masses of the distributions in (a), and the higher the curves are in (b). We found that custom interpolations of IS data using IDW and kriging (UKP, UK, and OK) out-performed the gridded datasets for both performance statistics, with IDW performing best overall. In agreement with these results, equivalent or superior performance of the IDW method relative to other interpolations including kriging and VP, specifically for hydroclimatological applications, has been observed in other regions as well^9, 42^. The best performing gridded dataset is PERSIANN, whose statistics in the study region more closely resemble those of custom interpolated datasets than other gridded products. The differences between performance statistic distributions are statistically significant (as evaluated by non-parametric two-sample Kolmogorov-Smirnov tests, see Supplementary Table 1), consistent across gauge densities (as illustrated in Fig. 6(b)), as well as consistent across location (as indicated by latitude) and basin size (see Supplementary Figure 8), and season (see Supplementary Figure 9). The rain gauge densities in (b) are 1998–2013 averages of basin-area daily densities according to the IS data; they do not directly pertain to gridded datasets, but they are indicative of gridded dataset input gauge densities because gridded product source data (used directly, or for calibration) also comes from Brazilian government agency sources.

## Discussion

The ‘data selection uncertainty’ problem identified here is similar to the ‘gigo’ (garbage in, garbage out) problem in modeling, but applied to regional data analysis. Although the need to base analyses and interpretation on high quality data appears self-evident, the inability to directly observe the true spatial process of interest at regional scales, and thus to *a priori* discriminate between a wide array of available or self-generated regional data products, means that regional data selection is not trivial. Instead, it should motivate environmental scientists to consider the state of practice in the field, with respect to the use of, and confidence placed in, the use of regional climatic data products. For example, in Northern Brazil, where we have identified significant and meaningful differences between rainfall datasets, a wide range of studies draw inference about historical climate patterns and trends^43^, drought^23^, the effects of land use change on hydrology^44, 45^, and relationships between hydroclimate and agriculture^46, 47^, *without* confronting data selection uncertainty. Our analyses suggest that the conclusions of these studies must be treated with caution, as the magnitudes of difference or trends within data products may be comparable to the magnitudes of difference between data products. Several studies in the region do explicitly address data selection uncertainty: by correlating rainfall and streamflow datasets and selecting the rainfall product with the greatest correspondence^48^, and by demonstrating that multiple rainfall products would generate similar results^49^. Overall, however, data selection uncertainty remains inconsistently acknowledged and unaccounted for by practitioners.

The empirical time series and signal-processing methodology used here (i.e. performance statistics) offers an approach to evaluate rainfall data quality for hydrological purposes across multiple river basins and at large spatial scales and is arguably an improvement on the state of practice for regional hydrology. Traditional rainfall data error estimation frameworks infer rainfall data quality at points using cross-validation methods, or over river basin areas based on runoff predictions made via a model^33, 41^. Point-scale evaluations do not address areal-scale data quality, and at regional scales - i.e. the 89 basin region in this study - a model-based approach would require 89 separate runoff model calibration/validation procedures, and would not generate results that are comparable between basins because the calibration error would be unique for each basin^10, 16^. Furthermore, the attribution of prediction error to calibration would be confounded with rainfall data input uncertainty. Lastly, the quality and reliability of rainfall-runoff model prediction relies on input stationary^50, 51^, which is not guaranteed in the study region due to climate and land use change. Thus, model-free approaches are desirable. Our empirical approach capitalizes on the relationships between variables (rainfall, streamflow) rather than on their exact values to evaluate rainfall dataset quality at basin scales. This method complements standard model-based evaluation, but is scalable and generalizable over large regions that challenge the use of models.

While it was possible to identify a best performing rainfall dataset based on streamflow correlation in this region, the results are likely to be site specific and specific to applications in which comparing rainfall signals to streamflow signals offers an appropriate test of quality. Evaluations should be made separately for new study areas, and potentially by comparison to reference datasets other than streamflow for different study purposes. For example, streamflow intercomparisons would not necessarily inform the suitability of a rainfall dataset for surface soil moisture estimation purposes, as would microwave remote sensing data. Similarly, interpolation methods such as UK (which can control for elevation) would likely improve upon IDW in mountainous areas. The differences between the datasets’ performance statistics were reduced when data were aggregated or smoothed over time, consistent with previous studies that have shown RS data to correspond well to IS data with greater temporal aggregation^52, 53^. Thus, at coarser temporal resolutions (monthly, annual), convenient gridded products remain attractive.

Critical climate change adaptation decisions are likely to derive from the understanding of emerging trends and variability in regional rainfall estimates. These results highlight the often-unacknowledged problem of ‘data-selection uncertainty’ in the detection and attribution of environmental change^37, 54^, and demonstrate a need for increased effort in quantifying this uncertainty and justifying data choice because analysts may reach divergent understandings due to data selection alone^55^. Identifying the often weak signals of change in noisy datasets is challenging, but analysts can reduce the uncertainty derived from data choice by (i) justifying dataset choices using selection methods such as the performance statistics demonstrated here, and/or (ii) including estimates of data-selection uncertainty (e.g. confidence intervals) in their findings. Evaluation of rainfall data prior to hydroclimatological analysis is both feasible (if streamflow records are available) and necessary. In contrast to the use of climate model outputs in analyses - where characterization of an ensemble of equally uncertain projections is best practice - if an individual dataset corresponds more closely with a reference of choice (e.g. streamflow) than other datasets, that dataset should be used for analysis.

## Methods

### Data

Gridded datasets include: Global Precipitation Climatology Project (GPCP) Version 1.2^56^; Climate Prediction Center (CPC) Unified Gauge-Based Analysis of Global Daily Precipitation Version 1 and RT data^26, 57^; Tropical Rainfall Measuring Mission (TRMM) 3B42 Version 7^58^; and Precipitation Estimation from Remotely Sensed Information using Artificial Neural Networks - Climate Data Record (PERSIANN-CDR) Version 1.1^36, 59^. GPCP, TRMM, and PERSIANN were acquired from public repositories via the IRI/LDEO Climate Data Library^60^ and CPC from the raincpc R package^61^. IS rainfall, streamflow, and geographic information systems (GIS) data were acquired from the Agência Nacional de Águas (ANA), and reservoir locations (used to select only unregulated river basins for analysis) from the Agência Nacional de Energia Elétrica (ANEEL). Of a total of 1,171 usable rain gauges in the study region, 942 were active (for varying durations) during the study period, and were used for analysis. Daily streamflow data was obtained for basins fully contained in the study region, gauged for at least a year, and with <10% of their area impacted by reservoirs. Analysis was based on 89 basins that met data quality criteria and overlapped with the interpolated rainfall region. All IS rainfall, streamflow, GIS data, and comprehensive documentation on data acquisition and quality assurance/quality control are provided in the “Curated rain and flow data for the Brazilian rainforest-savanna transition zone” data package^30^. Interpolated rainfall data are available upon request from the corresponding author. (See the Supplementary Discussion for more discussion of rainfall data).

We did not manipulate the spatial resolution of the daily rainfall datasets (all are obtained or generated at 0.25°, except for two sources at 0.5° and 1° resolution - see Table 1), nor did we compare the representation of rainfall based on spatial resolution. We did however briefly explore areal rainfall differences across rain gauge densities with respect to the known gauge densities of interpolated IS data - see Supplementary Figure 7). Typically, to compare rainfall datasets, one would aggregate (or disaggregate) rainfall datasets to a common grid using a method that conserves the total amount of rainfall in an area. Effectively, this study aggregates total daily rainfall to river basin units, without modifying original input data; this is done using a grid cell area-weighted mean of all cells located within a basin area, providing an unmodified representation of each datasets’ area-integrated rainfall over multiple basin scales, that is both conservative and representative of the practical needs of hydrologists and hydroclimatologists.

### Interpolation

We used four common and well-documented interpolation techniques^8, 15, 41, 42^: Voronoi (or Thiessen) Polygons (VP)^62^; Inverse-Distance Weighting (IDW)^63^, and Ordinary and Universal Kriging (OK, UK)^64, 65^. All interpolations were done on a 0.25° resolution grid. IDW and OK are local interpolations, for which we set the maximum interpolation distance (radius) to 300 km, an upper bound on estimated mean rainfall correlation distances in this region, which ranged between 100–300 km for IS and RS data, respectively. Rainfall correlation distances were estimated by fitting a semivariogram model^65^ to data on a random sample of 1,500 individual days (approximately 1/4 of the days in the full date range), and extracting semivariogram range estimates for each day. UK and UKP are ‘universal’ interpolations for the study region; their predictions rely on relationships established between predictor variables across the entire study region. Kriging methods can produce negative values, which were set to zero. To avoid edge effects in interpolations, the grid at which rainfall was interpolated is inset from the study region boundary by 100 km (the minimum mean correlation distance). For additional details on interpolation methods, see Supplementary Discussion.

### Trends and Hydroclimate Indices

For trend analyses, we used the non-parametric Seasonal Kendall test for monotonic trends in monthly total rainfall with correction for correlation between monthly blocks, and estimated the slope of the trend using the SK slope estimator^66, 67^; these are seasonally-adjusted modifications of the widely-used Mann-Kendall test^68^ and Theil-Sen’s slope estimator^69, 70^ that are targeted to hydrological time series.

Index values were calculated for each water year (October-September) in each basin, using river basin area average daily rainfall depths (mm/day) from all nine rainfall datasets, and streamflow depths (mm/day, which are basin-area normalized volumetric flow rates) at river basin scales. The index values recorded for an individual basin and rainfall dataset combination is the average of annual index values for that basin-dataset combination (there are 15 complete water years between 1998–2013). The runoff ratio (*RR*) is the simple ratio of total annual (water year) streamflow (*Q*) to total annual rainfall (*P*): *RR* = *Q*/*P*. Similarly, the evaporation ratio (*ER*) is the simple ratio of total annual (water year) evapotranspiration (*ET* = *P* − *Q*) to total annual rainfall (*P*): *ER* = *ET*/*P*. (Note that in these computations we assumed no deep percolation). Lastly, the Horton index (*HI*) is the ratio of evapotranspiration (*ET*) to available soil water (*W*): *HI* = *ET*/*W*, where soil water *W* = *P* − *Q*_*q*_, and *Q*_*q*_ is the direct runoff component of total flow (*Q*); *W* is equivalent to the sum of baseflow and *ET* - the total amount of water accessible to vegetation. Total flow was separated into baseflow and quickflow using a Lynne-Hollick recursive digital baseflow filter (three-pass, default parameter of 0.975)^71^. The Horton index is intended to be calculated over a growing season^35^, however, growing seasons vary across the river basins in this analysis, and many are year-round, thus the use of annual data.

### Performance statistics

Volumetric streamflow records were area-normalized and separated into baseflow and quickflow (direct runoff) using a Lynne-Hollick recursive digital baseflow filter (three-pass, default parameter of 0.975)^71^; the quickflow component can be more directly compared to rainfall. Both rainfall and quickflow time series were normalized to between 0 and 1. We identified the lag timescale (*τ*) that maximized the cross-correlation of rainfall and quickflow (the basin response timescale in units of days) for each basin, and lagged rainfall by *τ* for analysis of correlation and peak correspondence.

With respect to peak correspondence: we classified peaks in the normalized and lag-aligned rainfall and quickflow data by determining the position of peak extrema (observations that are preceded and followed by lower observations), as well as probabilities associated with peaks^72^. The probability associated with a peak quantifies the distinctness of the peak: more significant peaks are those surrounded by *several* lower observations. Peaks with lower probabilities are those that contain more information according to Kendall’s information theory^72, 73^. We call peaks with probabilities <0.05 ‘distinct’ (due to autocorrelation in the rainfall and flow time series, this is not a measure of statistical significance, but may nevertheless be used to distinguish more and less distinct peaks). ‘Peak correspondence’ is the rate at which distinct peaks in lagged rainfall match those in streamflow over a basin-specific response time window equivalent to 1/4 × *τ* (minimum = 1 day) (see Supplementary Figure 10). Correlation between the lagged rainfall and quickflow was assessed using non-parametric Spearman’s rank correlation^74, 75^. For more details, see the Supplementary Discussion.

### Code availability and computational tools

Code is available upon request from the corresponding author. We carried out all analyses and generated all figures within the Comprehensive R Archive Network (CRAN)^76^ programming environment (Version 3) on both Apple and Windows operating systems. See the Supplementary Discussion for a list of utilized software packages.

## Electronic supplementary material

Supplementary information
