# Supplementary material for: Addressing rainfall data selection uncertainty using connections between rainfall and streamflow
Source: Sci Rep. 2017 Mar 16;7:219. doi: 10.1038/s41598-017-00128-5 (PMC5427843; doi:10.1038/s41598-017-00128-5)
Supplement: Supplementary file 1 — Supplementary information [file 41598_2017_128_MOESM1_ESM.pdf]

Supplementary Information: Addressing rainfall data selection uncertainty using connections between rainfall and streamflow

Morgan C. Levy[1*], Avery Cohn[2], Alan Vaz Lopes[3] & Sally E. Thompson[4]

[1*] Energy and Resources Group, University of California, Berkeley, USA

[2] Fletcher School, Tufts University, USA

[3] National Water Agency (ANA), Brazil

[4] Department of Civil and Environmental Engineering, University of California, Berkeley, USA

Figures

All figures were generated using the Comprehensive R Archive Network (CRAN)[1] programming environment (Version 3) on both Apple and Windows operating systems. See the Supplementary Discussion for a list of utilized software packages. See the Methods section of the main text for data source references.

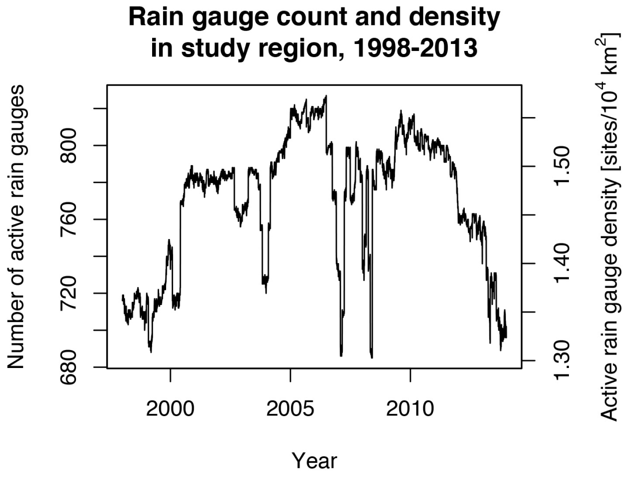

Figure 1: **Rain gauge counts and densities in study region.** The number of active rain gauges (left vertical axis) and active rain gauge density (right vertical axis), daily between 1998-2013, in the eight-state and 5.25 million km^2 study region. Rain gauge data is from the curated data package used in this study.[2]

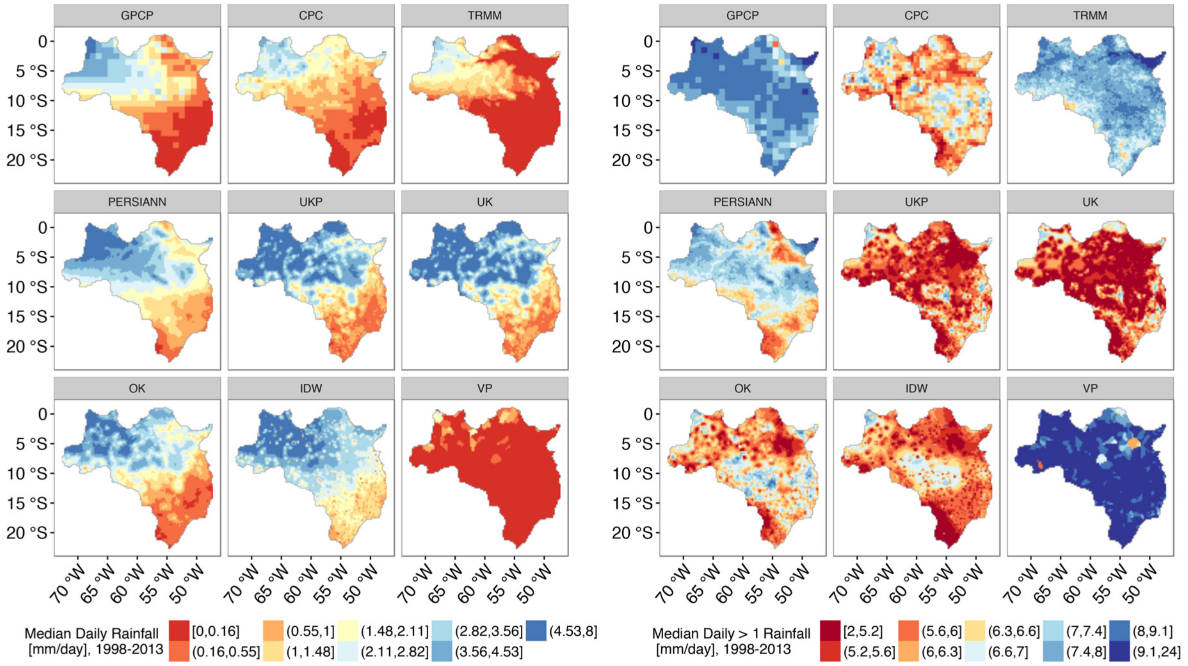

Figure 2: **Median and wet-day median rainfall.** Median daily and wet-day (days with ≥ 1 mm/day) median daily rainfall between 1998-2013 over the study region according to different datasets. Median values were calculated at each 0.25° grid cell. These maps were generated in R, Version 3 (https://cran.r-project.org/).[1]

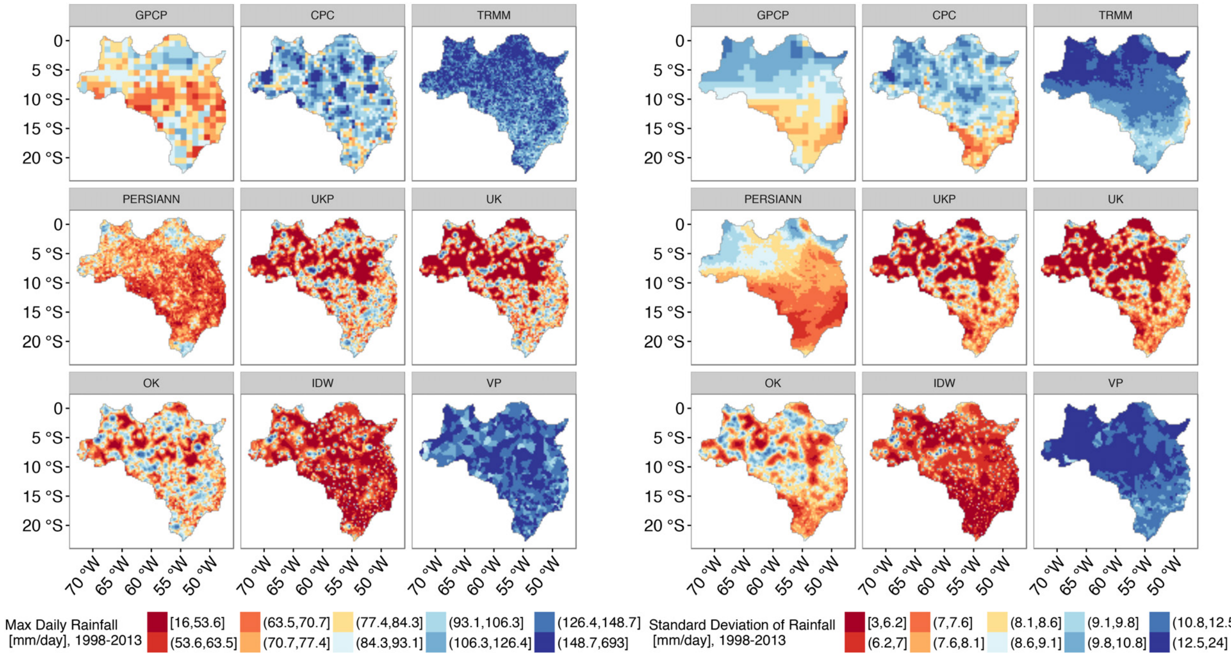

Figure 3: **Extremes and variability of rainfall.** Maximum (left) and standard deviation (right) of daily rainfall between 1998-2013 over the study region according to different datasets. Maxima and standard deviations were calculated at each 0.25° grid cell. These maps were generated in R, Version 3 (https://cran.r-project.org/).[1]

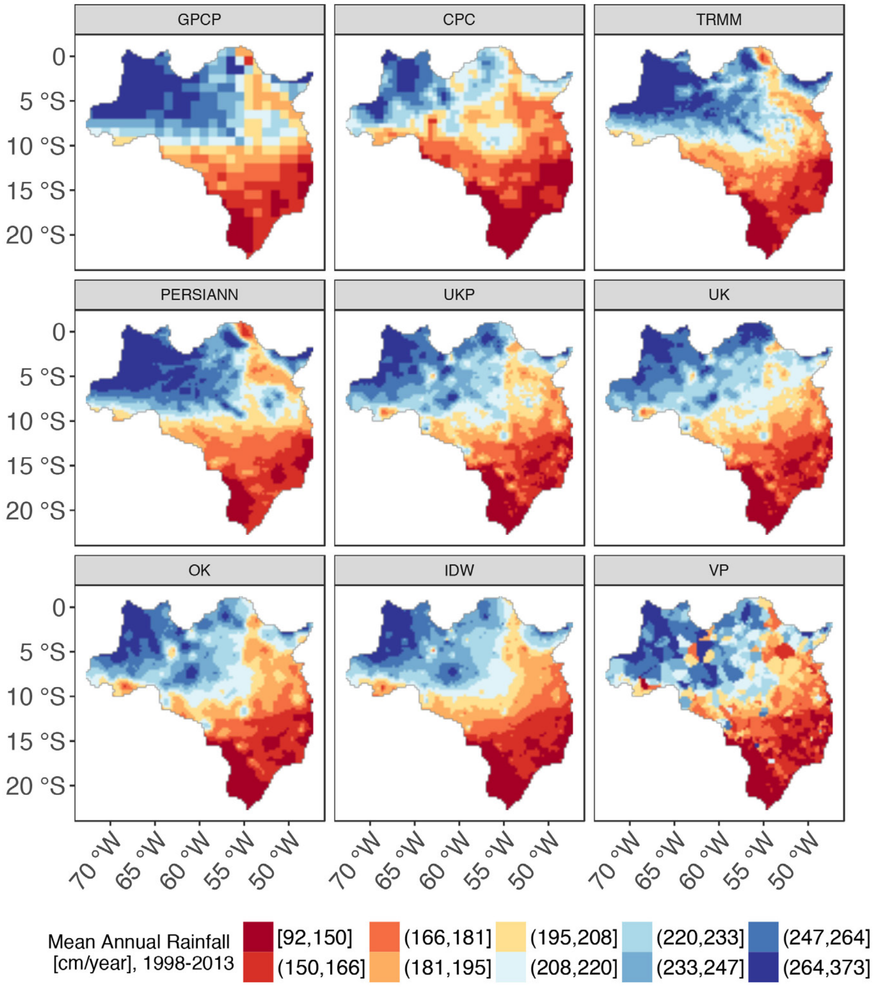

Figure 4: **Mean annual total rainfall.** Mean annual water year (October-September) totals of rainfall between 1998-2013 over the study region according to different datasets. Mean values were calculated at each 0.25° grid cell. These maps were generated in R, Version 3 (https://cran.r-project.org/).[1]

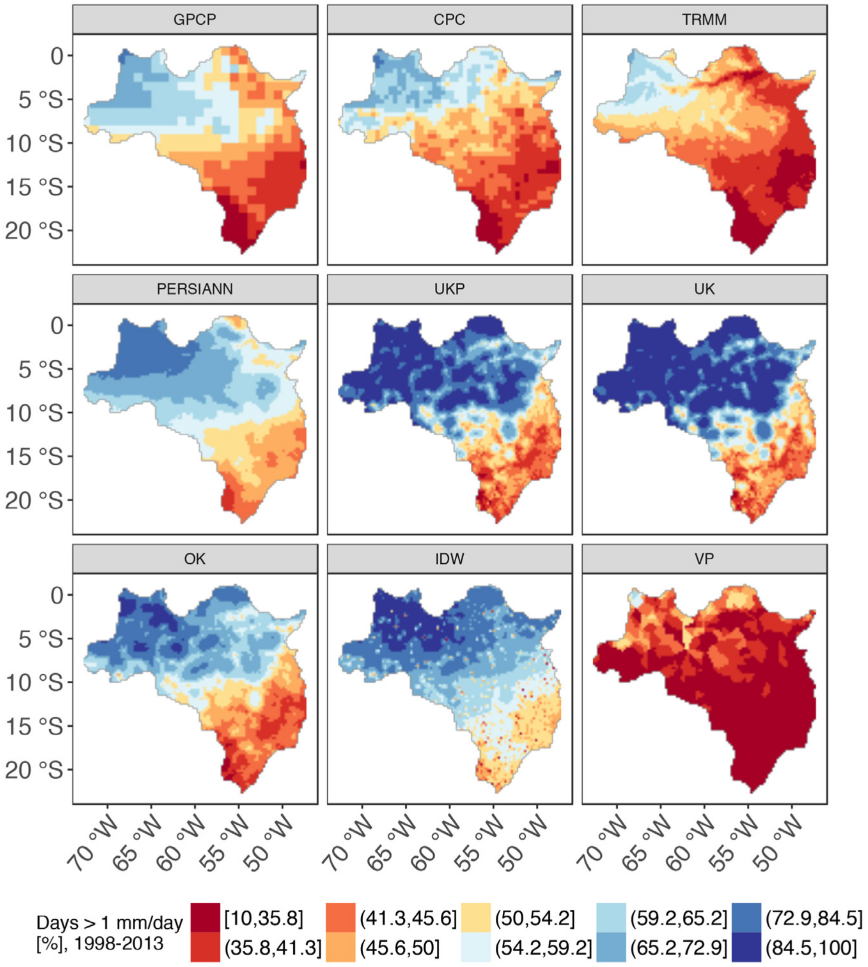

Figure 5: **Occurrence of rainfall.** Daily rainfall occurrence (percent of wet days, when rainfall depths are $\geq$ 1mm) between 1998-2013 over the study region according to different datasets. Occurrence was calculated at each 0.25° grid cell. These maps were generated in R, Version 3 (https://cran.r-project.org/).[1]

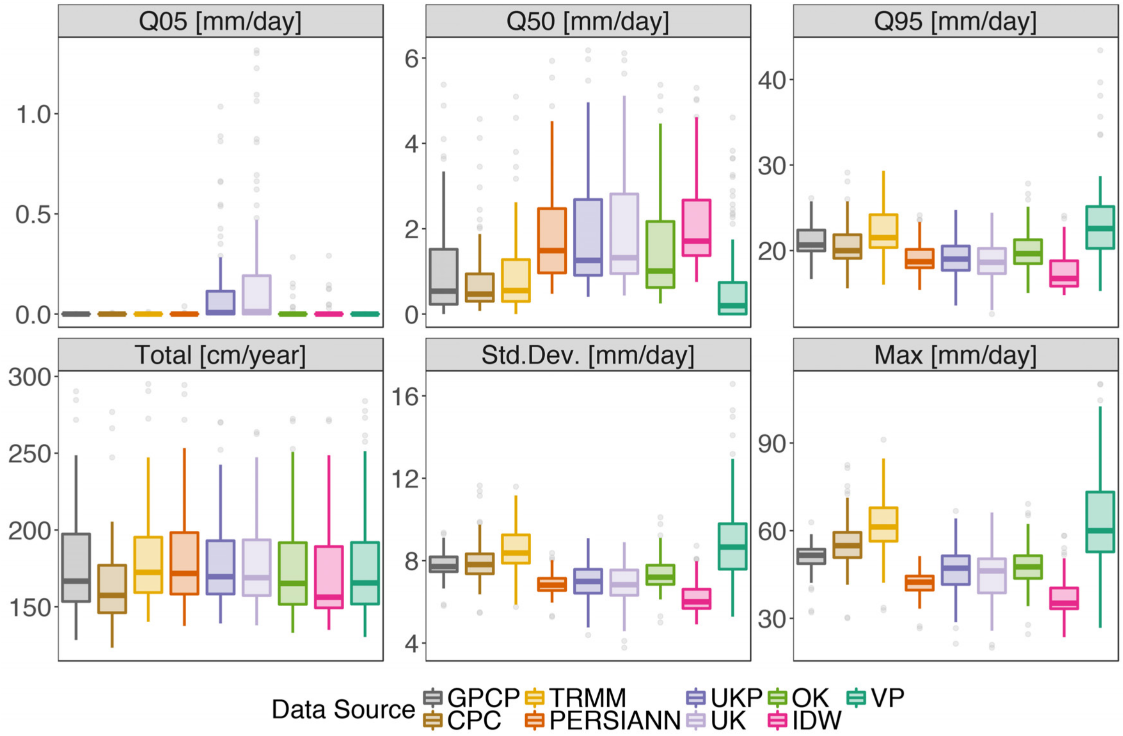

Figure 6: **Supplemental daily rainfall statistics in river basins according to different rainfall datasets.** From left to right, top to bottom, the panels show the 5th, 50th (median), and 95th percentiles of daily rainfall (mm/day); the (mean) total annual rainfall (cm/year); standard deviation of daily rainfall (mm/day); and the (mean annual) maximum daily rainfall (mm/day). Each boxplot is generated with n=89 (river basin) statistic results, calculated using basin area-average rainfall from the given rainfall dataset (colors) from all days between 1998-2013.

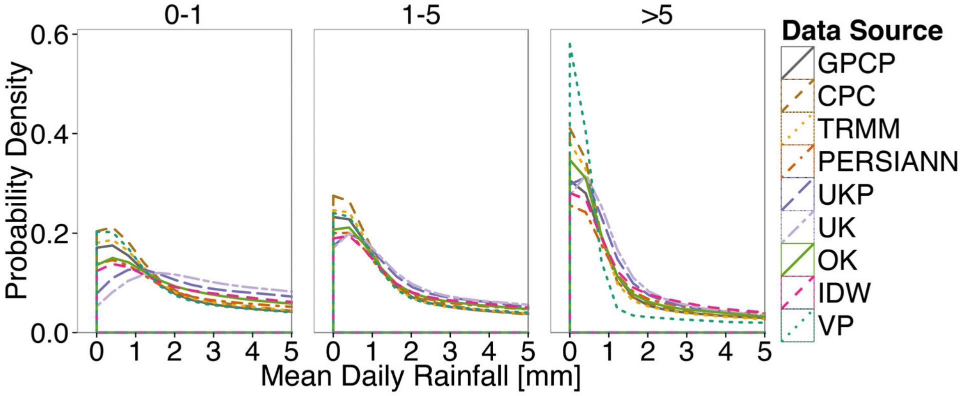

Figure 7: **Daily rainfall distributions by rain gauge density.** Empirical probability distributions of daily rainfall between 1998-2013 across sample areas in the study region by rainfall dataset (curves) and rain gauge density bins (panels). Rain gauge density bins are labeled on the top of panels, and refer to the number of rain gauges per 10^4 km^2. Samples at different gauge densities are from rainfall-averaging areas extending outward from 100 regularly-sampled points in the study region with radii between 10-200 km. The number of observations in each gauge density bin was balanced by sub-sampling due to a greater number of observations in the low-density bin: each final bin was composed of 10^6 observations. Distributions deviate between datasets primarily at the lowest and highest rain gauge densities. GPCP, CPC, TRMM, and VP report a greater number of dry-day, low-rainfall depth events (< 1 mm/day) than UKP, UK, OK, IDW, and PERSIANN, especially at lower and higher gauge densities. The latter group of datasets register wet-day (≥ 1 mm/day), medium intensity rainfall depths more frequently than the first group, especially at the lowest and highest rain gauge densities.

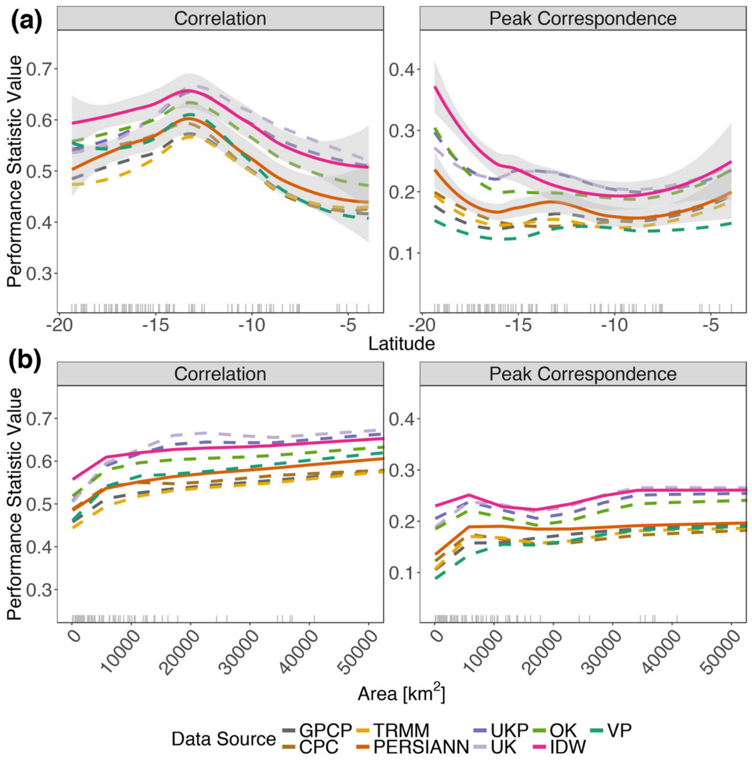

Figure 8: **Differences in rainfall data quality as indicated by performance statistics, by latitude and river basin area.** Panel (a) shows performance statistics (correlation and peak correspondence) by rainfall dataset (colors) plotted as local regression-smoothed curves across the range of latitudes (at basin centroids) in the study region, with 95% uncertainty intervals (shaded). Panel (b) shows the same performance statistic curves plotted across the range of river basin area sizes in the study region. In both panels, solid lines indicate the best-performing gridded (PERSIANN) and custom-interpolated (IDW) datasets. In (a), non-overlapping uncertainty intervals indicate distinguishable performance between datasets; dashed-lines indicate all other datasets with uncertainty intervals that are not displayed, but are of similar width. In (b), uncertainty intervals overlap in most of the displayed range due to the sparse sampling of basin size across the range of basin sizes, and are therefore not shown. Gray tick-marks at the bottom illustrate the spread of latitudes and rain gauge densities in the 89 river basins.

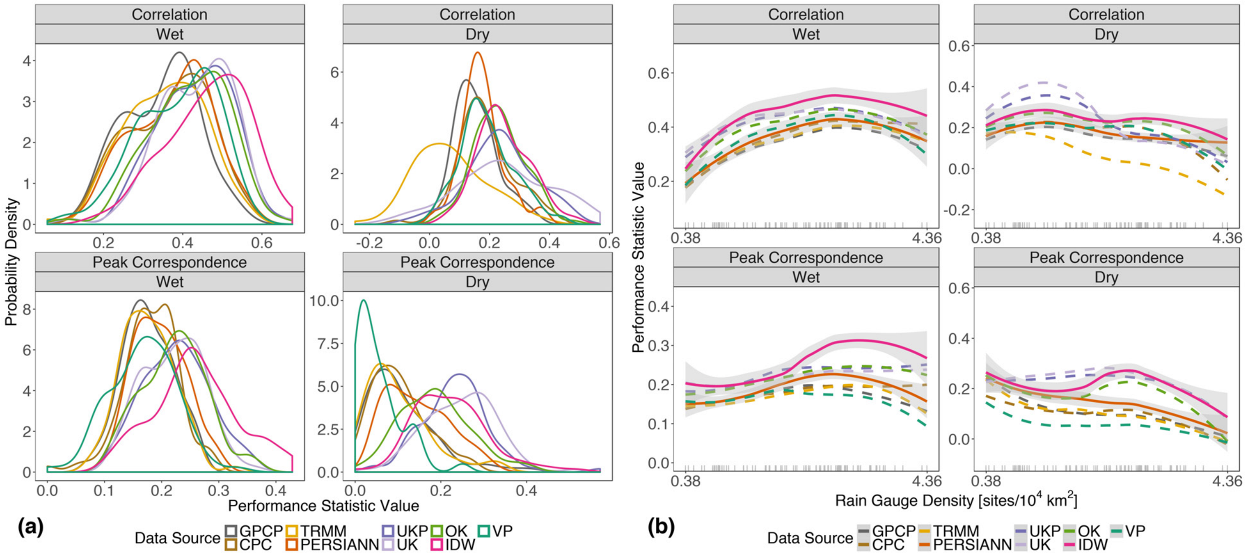

Figure 9: **Differences in rainfall data quality as indicated by performance statistics, by season.** Panel (a) shows kernel-smoothed empirical probability distributions of performance statistics (correlation and peak correspondence) by rainfall dataset and season (wet: October - April, dry: May - September). Panel (b) shows the same performance statistics plotted by season as local regression-smoothed curves across the range of rain gauge densities in the study region, with 95% uncertainty intervals (shaded). In (b), solid lines and shaded regions indicate the best-performing gridded (PERSIANN) and custom-interpolated (IDW) datasets and their 95% uncertainty intervals, respectively; non-overlapping uncertainty intervals indicate distinguishable performance between datasets; dashed-lines indicate all other datasets with uncertainty intervals that are not displayed, but are of similar width; gray tick-marks at the bottom illustrate the spread of rain gauge densities in the 89 river basins.

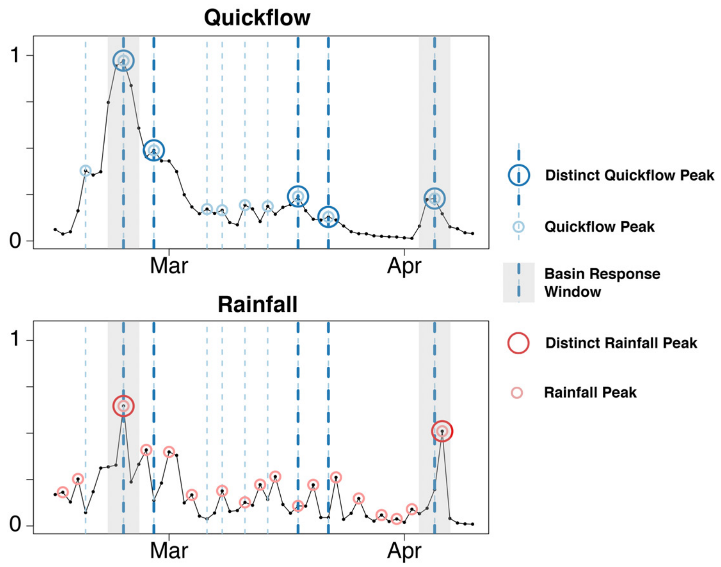

Figure 10: **Schematic of rainfall and streamflow peak correspondence methodology.** Daily quickflow (top) and rainfall (bottom) for a two-month period; the data have been standardized to [0,1]; rainfall has been lagged to maximize rain and flow cross-correlation. 'Peak correspondence' is a performance statistic that measures the rate at which distinct rainfall peaks correspond to distinct quickflow peaks within a basin-specific response time window. The shaded windows illustrate cases where distinct peaks correspond; on the left - an exact match, and on the right - a match within the window.

Tables

**Statistic**	**Data Source**	*GPCP*	*CPC*	*TRMM*	*PERSIANN*	*UKP*	*UK*	*OK*	*IDW*
Correlation	*CPC*	**0.034**							
	TRMM	0.3	**0.008**						
	PERSIANN	0.078	0.988	**0.008**					
	UKP	**0**	**0.003**	**0**	**0.003**				
	UK	**0**	**0.001**	**0**	**0.001**	0.948			
	OK	**0**	**0.003**	**0**	**0.001**	0.868	0.222		
	IDW	**0**	**0**	**0**	**0**	0.113	0.16	**0.034**	
	VP	**0.014**	0.868	**0.001**	0.948	**0.005**	**0.002**	**0.008**	**0**
Peak Correspondence	*CPC*	0.3							
	TRMM	0.756	0.3						
	PERSIANN	**0.003**	0.16	**0.003**					
	UKP	**0**	**0**	**0**	**0**				
	UK	**0**	**0**	**0**	**0**	0.988			
	OK	**0**	**0**	**0**	**0**	0.113	0.078		
	IDW	**0**	**0**	**0**	**0**	0.16	0.3	**0.005**	
	VP	**0.022**	**0.003**	0.052	**0**	**0**	**0**	**0**	**0**

Table 1: Two-sample Kolmogorov-Smirnov test p-values (rounded to three decimal places) for differences in distributions of performance statistics (for year-round data). Datasets with performance statistic distributions that are significantly different from each other are bolded (p-value < 0.05).

Supplementary discussion

Additional details about rainfall data

Rain gauge density factors into the quality of gridded datasets to the extent that each dataset relies on gauge data: RS datasets (GPCP, TRMM, PERSIANN) rely on gauge data for calibration, and the gridded IS dataset (CPC) relies on gauge data entirely. There is overlap in the gauge data used in custom interpolations (from the custom data package[2]) and quality-controlled data released by the Brazilian government and included in the National Center for Atmospheric Research (NCAR) Global Precipitation Climatology Centre (GPCC) monthly product, which is used to calibrate all three RS products evaluated in this study. The exact number of overlapping gauges across all gridded and custom interpolated products is not available as data creators do not release detailed source data. Based on information provided in referenced source documents (see Methods), our custom IS interpolations incorporate gauged locations that are additional to those used in the gridded datasets, and incorporate higher temporal resolution gauge data directly (instead of inclusion of only monthly IS data for calibration, such as in the RS products[3]). Therefore it is possible that the quality of custom IS interpolations relative to gridded datasets (according to performance statistics) derives primarily from the greater number of rain gauges used in the IS interpolations. Confirmation of this assertion for gridded RS datasets (GPCP, TRMM, PERSIANN) would require an ability to separate the contribution of satellite retrievals (and associated processing algorithms) from the contribution of rain gauge calibration to the overall product; for the gridded IS dataset (CPC), confirmation would require use of the CPC interpolation method on the custom data. These tasks are beyond the scope of this study, and therefore

we do not answer this question.

Our custom interpolations of IS data provide estimates of total rainfall amounts that are similar to gridded data (see Supplementary Figures 4 and 6), especially when aggregated up to monthly time scales. No single dataset reports consistently higher or lower rainfall depths at any given location. According to statistics calculated over large to small sample areas (100 regularly-sampled locations across the study region with radii of 200 km and 10 km, respectively): IDW, OK, and UKP report the lowest proportion of extreme rainfall (highest and lowest value) and anomalous rainfall (relative to mean of all datasets combined) at daily, monthly, and annual time scales. All gridded datasets and VP, and in some cases UK, are responsible for a greater fraction of extreme and anomalous rainfall estimates (although no single dataset is responsible for $> 50\%$). The datasets providing the most extreme/anomalous estimates (and the percentage of extremes/anomalies they are responsible for) change with respective to the temporal resolution (daily, monthly, annual). According to cumulative sum plots, seasonality is consistent across the different datasets.

Selection of IS interpolation resolution

In an analysis of rain gauge (pair) distances across the study region between 1998-2013, the average median distance is 1,150 km; the average 5% quantile distance is 250 m, and the average 95% quantile distance is 2,370 km. While the average minimum distance is 2km, this distance occurs for only a handful of rain gauge locations. Across the whole study region, most areas have rain gauges located at a distance of greater than 1,000 km, for which a 0.25° (28 km) resolution is sufficient. In a preliminary analysis, we carried out local IS interpolations over basin areas at a 0.05° resolution; our analysis with those interpolations yielded equivalent results, thus the performance of IS interpolations relative to RS data is not a function of interpolation resolution.

Selection of IS interpolation specifications

We selected IDW parameters, UK predictor variables (latitude, longitude, elevation,[4] and RS data - PERSIANN), and compared prediction error of the different interpolation methods and UK specifications using k-fold cross validation (CV). CV results were evaluated using correlation, error interquartile ranges, and RMSE of the errors normalized by standard deviation of observations. Kruskal-Wallis tests confirmed that these metrics discriminated between the interpolation methods' performance. An IDW parameter of 1.5, and UK (UKP) covariates of latitude, longitude, and elevation (and PERSIANN) were best performing. The UKP interpolation we used combines RS and IS data, which has been recommended.[5,6]

Additional information on hydroclimate indices

Normality tests (q-q plots and the Shapiro-Wilk test[7]) suggest that the runoff ratio and Horton index values (for each rainfall dataset) and/or means (across rainfall datasets) may be non-normal, demonstrating that alternative methods for the calculation of confidence intervals (e.g. non-parametric bootstrap sampling) may be preferred.

Additional information on performance statistics

IDW performs best on average, but the universal kriging methods (UKP and UK) can outperform IDW at low gauge densities. Unlike IDW and OK, which are local interpolations, UKP and UK generate mean rainfall values at locations with no nearby rain gauges (using 'universal' predictors such as elevation across the full study region), thereby increasing the occurrence of nonzero rainfall values at those locations. Because a greater number of nonzero rainfall values will correspond with flow rises (relevant to correlation) and peaks (relevant to peak correspondence) more frequently, kriging methods demonstrate better performance at very low gauge densities. Therefore, this result does not necessarily imply better representation of rainfall by kriging methods in low gauge density areas. Lastly, the kriging method incorporating RS data (UKP) performs no better than its IS-only counterpart (UK), although inclusion of the RS data in UKP attenuates UK's otherwise higher median daily values and occurrence.

With respect to the peak correspondence response time windows: in windows exceeding $1/3 \times \tau$ (τ is the basin response timescale in units of days), the peak correspondence method fails to distinguish between datasets. Use of a static window of 1 and 2 days across all basins yielded similar results to those presented in the main text (where a basin-specific window of $1/4 \times \tau$ was used). In the context of a contingency or error matrix analysis,[8] peak correspondence is the true-positive rate (TPR) at which distinct peaks match within a window of time that accounts for potential mismatches in the estimated basin response time. Thus, 1-TPR gives a 'false-negative' rate, corresponding to type-II error, or error of omission, in the rainfall datasets.

The performance statistics were validated on an external set of rainfall and streamflow data from seven Australian basins of various sizes, including those with yearlong (wet) and intermittent (dry) flow of different periods of record (2 - 39 years), provided in the `R` `hydromad` package.[9] Real rainfall from each basin was perturbed with normally-distributed additive random noise (mean = 0, standard deviation equal to a range of between 0.25 to 2 times the rainfall standard deviation) to create 100 synthetic rainfall datasets per basin; negative values of perturbed rainfall were set to zero, thus synthetic rainfall included additional rainfall events, as well as eliminated existing ones. The generation of 100 synthetic datasets was repeated 100 times (iterations) for each of the Australian basins. For each of the 100 iterations for each basin, we calculated the

percentage of times each performance statistic (correlation and peak correspondence) correctly identified the real rainfall dataset from the combined set of synthetic (100) and real (1) datasets. Correlation and peak correspondence reliably selected the real rainfall dataset across basins with different flow types (intermittent and yearlong) and time series lengths; correlation was, however, better able to distinguish the correct dataset when random perturbations were small (low signal to noise ratios). When the standard deviation was set to 1-2 times the real rainfall standard deviation, both performance statistics correctly identified the real rainfall dataset 100% of the time. When the standard deviation was smaller, for example, 0.5 times the rainfall standard deviation, correlation identified the correct rainfall dataset again 100% of the time, however peak correspondence identified the correct rainfall dataset on average (over all basins) 79% of the time. Specifically, peak correlation identified the correct rainfall dataset in two of the seven basins 100% of the time, regardless of the signal to noise ratio. However, it identified the correct rainfall dataset with decreasing accuracy in other basins as the signal to noise ratio was decreased. There were no similar features (e.g. size, intermittency) in the basins for which peak correspondence worked best or worst (although short record durations negatively affect peak correspondence due its reliance on peak events, which may occur infrequently, especially in dry basins). Thus, we assume that variation in the ability of peak correspondence to identify the correct rainfall dataset has to do with the nature of runoff data from individual basins; basins in which peak correspondence is most likely to work well, given low signal to noise ratios, are those that produce clear (and ideally, frequent) quickflow response signals.

We also evaluated a suite of alternative performance statistics, including: mutual information;[10,11] peak correspondence calculated with less distinct (higher probability) peaks (e.g. using all, instead of just distinct peaks); normalized cumulative distances; peak correspondence and correlation with respect to local regression-smoothed rainfall time series; peak correspondence and correlation with respect to probability- and information-weighted quickflow and rainfall peaks; and moving-window correlation of peak magnitudes for rainfall and quickflow. Mutual information results were similar to correlation, but were less reliable in identifying true rainfall when tested on the suite of external data (described above), as were peak correspondence measures calculated with less distinct peaks. The remaining trial statistics either failed to reliably identify the correct rainfall dataset in validation tests, or duplicated the results of the two primary performance statistics.

Software

Formatting and analysis of spatial data relied on the core R spatial analysis packages `sp`,[12,13] `raster`,[14] `gstat`[15] and their dependencies. To estimate delay times between rainfall and flow events and obtain test

data for performance statistic validation, we used the `hydromad` package.[9] To perform baseflow and quickflow separation, we used the `hydrostats` package.[16] To categorize peaks in rainfall and quickflow, and calculate their probability and significance, we used the `pastecs` package.[17] For construction and analysis of error matrices, we used the `caret` package.[18] We used the `rkt` package[19] for Seasonal Kendall (SK) tests and trend estimators. Figures and maps were generated using using the `ggplot2`,[20] `ggmap`,[21] and `globe`[22] packages and their dependencies.

References

[1] R Core Team. *R: A Language and Environment for Statistical Computing* http://www.R-project.org/ (2015) (Date of access: 07/01/2015).

[2] Levy, M. C. *Curated Rain and Flow Data for the Brazilian Rainforest-Savanna Transition Zone* http://www.hydroshare.org/resource/e82e66572b444fc5b6bf16f88f911f77 (Consortium of Universities for the Advancement of Hydrologic Science, Hydroshare, 2016) (Date of access: 12/03/2016).

[3] Gehne, M., Hamill, T. M., Kiladis, G. N. & Trenberth, K. E. Comparison of global precipitation estimates across a range of temporal and spatial scales. *Journal of Climate* 10.1175/JCLI–D–15–0618.1 (2016, in press).

[4] Jarvis, A., Reuter, H., Nelson, A. & Guevara, E. Hole-filled SRTM for the globe Version 4, available from the CGIAR-CSI SRTM 90m Database http://www.cgiar-csi.org/data/srtm-90m-digital-elevation-database-v4-1 (2008) (Date of access: 18/10/2014).

[5] Scheel, M. L. M. *et al.* Evaluation of TRMM Multi-satellite Precipitation Analysis (TMPA) performance in the Central Andes region and its dependency on spatial and temporal resolution. *Hydrology and Earth System Sciences* **15**, 2649–2663 (2011).

[6] Teng, H., Shi, Z., Ma, Z. & Li, Y. Estimating spatially downscaled rainfall by regression kriging using TRMM precipitation and elevation in Zhejiang Province, southeast China. *International Journal of Remote Sensing* **35**, 7775–7794 (2014).

[7] Shapiro, S. S. & Wilk, M. B. An analysis of variance test for normality (complete samples). *Biometrika* **52**, 591–611 (1965).

[8] Fawcett, T. An Introduction to ROC Analysis. *Pattern Recogn. Lett.* **27**, 861–874 (2006).

[9] Andrews, F. T., Croke, B. F. W. & Jakeman, A. J. An open software environment for hydrological model assessment and development. *Environmental Modelling & Software* **26**, 1171–1185 (2011).

[10] Shannon, C. E. & Weaver, W. *The mathematical theory of communication.* (University of Illinois Press, Urbana, 1949).

[11] Cover, T. M. & Thomas, J. A. *Elements of information theory* (Wiley, New York, 1991).

[12] Bivand, R. S., Pebesma, E. & Gomez-Rubio, V. *Applied spatial data analysis with R, Second edition* (Springer, NY, 2013).

[13] Pebesma, E. J. & Bivand, R. S. Classes and methods for spatial data in R. *R News* **5**, 9–13 (2005).

[14] Hijmans, R. J. *raster: Geographic Data Analysis and Modeling, Version 2.4-18* http://CRAN.R-project.org/package=raster (2015) (Date of access: 21/09/2015).

[15] Pebesma, E. J. Multivariable geostatistics in S: the gstat package. *Computers & Geosciences* **30**, 683–691 (2004).

[16] Bond, N. *hydrostats: Hydrologic indices for daily time series data, Version 0.2.3* http://CRAN.R-project.org/package=hydrostats (2014) (Date of access: 26/09/2015).

[17] Grosjean, P. & Ibanez, F. *pastecs: Package for Analysis of Space-Time Ecological Series, Version 1.3-18* http://CRAN.R-project.org/package=pastecs (2014) (Date of access: 26/09/2015).

[18] Wing, M. K. C. f. J. *et al.* *caret: Classification and Regression Training, Version 6.0-52* http://CRAN.R-project.org/package=caret (2015) (Date of access: 29/09/2015).

[19] Marchetto, A. *rkt: Mann-Kendall Test, Seasonal and Regional Kendall Tests, Version 1.4* http://CRAN.R-project.org/package=rkt (2015) (Date of access: 20/10/2015).

[20] Wickham, H. *ggplot2: Elegant Graphics for Data Analysis* http://ggplot2.org (Springer-Verlag New York, 2009) (Date of access: 30/09/2015).

[21] Kahle, D. & Wickham, H. ggmap: Spatial Visualization with ggplot2. *The R Journal* **5**, 144–161 (2013).

[22] Baddeley, A. & Lawrence, T. *globe: Plot 2D and 3D Views of the Earth, Including Major Coastline, Version 1.1-2* https://CRAN.R-project.org/package=globe (2016) (Date of access: 07/09/2016).
